# Planetary health education in Indian medical curricula

**DOI:** 10.1016/j.joclim.2025.100481

**Published:** 2025-07-05

**Authors:** Prasoon Pattanaik, Anandita Pattnaik

**Affiliations:** a Department of Community Medicine, Srirama Chandra Bhanja Medical College and Hospital, Cuttack, Odisha, India; b Current Regional Lead (India), Planetary Health Report Card, India; c Policy Officer, UK Health Alliance on Climate Change, ℅ BMJ Publishing Group, Tavistock Square, London, UK; d Former Regional Lead (India), Planetary Health Report Card, India

**Keywords:** Planetary health, Medical education, India, Sustainability, Interdisciplinary research, Climate change

## Abstract

**Introduction:**

This study assesses the state of planetary health education in Indian medical schools and proposes actionable recommendations for enhancement.

**Materials and Methods:**

The Planetary Health Report Card (PHRC), an international student-driven metric-based tool, was used to evaluate planetary health content in six report cards from four prominent Indian medical schools. Five key categories were evaluated: planetary health curriculum, interdisciplinary research, community outreach and advocacy, support for student initiatives, and campus sustainability measures.

**Results:**

Disparities were found in integrating planetary health into medical education among surveyed schools. While some topics like "the effect of extreme heat and pollution on health" were commonly integrated, critical topics such as "the carbon footprint of healthcare systems" were often overlooked. Limited interdisciplinary research and community engagement were noted, with insufficient support for student initiatives. However, all schools showed progress in campus sustainability. The National Medical Commission’s curriculum includes only limited content on planetary health. The absence of dedicated coursework and inconsistent implementation has resulted in uneven integration across the country, with much of it depending on individual faculty initiatives.

**Conclusion:**

Recommendations include developing dedicated coursework with clear learning outcomes, promoting planetary health research, organizing outreach activities, making educational materials accessible, supporting student initiatives, and enhancing campus sustainability. These recommendations aim to equip future healthcare professionals with the knowledge and skills to address the relationship between human health and planetary well-being, fostering a sustainable healthcare system in India.

## 1 Introduction

The concept of planetary health is a transdisciplinary field focused on the impacts of human disruptions to Earth's systems on health [1,2]. It examines the environmental determinants of health alongside other wider determinants such as poverty, nutrition, water, energy, and urbanization [3].

Human health depends on a stable climate and biodiverse ecosystems [4]. Despite medical advances, climate change and ecological degradation are major public health threats, driving air pollution, extreme weather, infectious disease spread, food and water insecurity, non-communicable diseases, and mental health issues [5]. Medical curricula must urgently adapt to prepare future doctors for these challenges [6].

Only 15 % of medical schools worldwide included climate change in their teaching according to a study in 2019–20 [7]. The Planetary Health Report Card (PHRC), developed in 2019, evaluates medical institutions on their planetary health initiatives and has spread to over 150 schools globally [8]. In India, one study reported that 70 % of medical colleges lack formal inclusion of planetary health topics in their curricula [9]. As future healthcare practitioners, Indian medical graduates must be equipped to address the health impacts of environmental change to build climate-resilient health systems. This study assesses the planetary health content currently included in a small sample of public and private medical colleges in India.

## 2 Methodology

The PHRC is an international student-driven metric-based tool that aims to evaluate health professional schools on discrete metrics regarding planetary health education [10]. In this study, six report cards from four Indian medical schools were analyzed: Srirama Chandra Bhanja Medical College and Hospital (SCBMCH) [11,12], St. John’s Medical College (SJMC) [13,14], Father Muller Medical College (FMMC) [15], and Kalinga Institute of Medical Sciences (KIMS) [16].

SCBMCH participated in all three years of the study (2022, 2023, and 2024), SJMC in two non-consecutive years (2022 and 2024), and FMMC and KIMS participated only in the final year (2024). All six report cards were analyzed to enable vertical and horizontal comparisons. In each school, a team of student representatives, consisting of a leader and at least one other student, carried out an assessment of courses offered at their respective institutions, employing the PHRC template. Ethics exemption was obtained from the Institutional Ethics Committee of each school.

Students used a combination of publicly available data (e.g. school websites), student experiences, and faculty input to complete the PHRC tool, helping to capture planetary health content that may not have been formally documented. The PHRC assigns grades across five sections: curriculum, research, community engagement, student initiatives, and campus sustainability. Each section is scored based on specific metrics, with raw scores converted into percentages. A weighted average is then calculated to determine the overall institutional grade, with the curriculum section given greater weight due to its broader scope. Letter grades A, B, C, D, and F are assigned based on predefined percentage ranges to standardize institutional performance in planetary health. Scores within the top 5 % of a grade range receive a “+”, while those in the bottom 5 % receive a “–” [[11], [12], [13], [14], [15], [16]]. To maintain consistency across all participating institutions on a global scale, each report card underwent evaluation by a regional lead not affiliated with the institution in question. To minimize personal bias, an approach where a group of students assessed the curricula being taught in the previous year of medical education, along with faculty feedback, was implemented.

## 3 Results

Results focus on each specific area of the PHRC metrics [17]. To compare how the four medical colleges perform across various metrics in the year 2023–24, refer to Table 1.

**Table 1:** Comparison of the 4 Indian medical schools across various PHRC metrics in the year 2023–24.

Metric	SCBMCH	SJMC	FMMC	KIMS
Overall grade	C	C	D	C+
Elective Courses on Planetary Health	No	Yes, multiple electives	No	No, some electives include relevant lectures
Extreme Heat, Health Risks, Climate Change	In-depth	Briefly covered	Briefly covered	In-depth
Impact on Marginalized Populations	Briefly covered	Briefly covered	Not covered	Briefly covered
Healthcare System Carbon Footprint	Not covered	Not covered	Not covered	Not covered
Faculty Overseeing Planetary Health Integration	No	Yes	No	Yes
Planetary Health Researchers	Individual faculty (not primary focus)	Faculty (primary focus)	Individual faculty (not primary focus)	Individual faculty (not primary focus)
Dedicated Department for Planetary Health Research	Yes	No	No	Yes
Planetary Health Conferences (in past 3 years)	Yes	Yes	None	Yes
Community-Facing Planetary Health Events	Annually	Annually	No	Annually

### 3.1 Integration of planetary health into the medical curriculum

The National Medical Commission (NMC) curriculum includes planetary health topics such as the health impacts of extreme heat, pollution, climate-related disease spread, and its disproportionate effects on marginalised populations [12,18]. Still, the extent to which planetary health topics were incorporated into the curricula varied significantly across the universities examined. SCBMCH and KIMS included content that covered the effect of extreme heat and pollution on health by examining heat-related conditions like heat stroke and heat stress in environmental modules [11,16]; however, FMMC and SJMC offered limited coverage. *The* impact of climate change on marginalised groups was examined in SCBMCH through environmental impacts on tribal communities, while SJMC and KIMS offered brief discussions [11,16] and FMMC lacked this topic. Only SJMC introduced electives covering topics like climate change, sustainability and air pollution [13,14] and while SJMC and KIMS had faculty specifically involved in planetary health, SCBMCH and FMMC did not [11,13]. Finally, none of the schools covered healthcare's carbon footprint [17].

These differences are particularly notable given that all the institutions follow the NMC curriculum, which is intended to be uniform across medical schools in India. The absence of dedicated coursework and standardized implementation has led to inconsistent integration across institutions. As seen above, the inclusion of key planetary health concepts often depends on individual faculty interests and institutional priorities, resulting in significant variability in addressing these topics.

### 3.2 Interdisciplinary planetary health research

SCBMCH, KIMS, and FMMC had faculty involved in planetary health research, though not as a primary focus [11,15,16]. SJMC faculty were notably active, with publications emphasizing planetary health education [13]. SCBMCH's Utkal University and KIMS’s KIIT University had departments conducting related research but lacked integration for medical students [11,16].

SCBMCH held seminars on climate change at the university level, but these lacked health interlinkages, and the participation of medical students was missing. SJMC co-hosted two Bioethics conferences while KIMS hosted conferences on planetary health [11,13,16]. KIMS maintained a website with updates related to health and the environment, whereas SCBMCH, SJMC and FMMC lacked dedicated websites to planetary health [11,13,15,16,19,20]. All schools scored poorly on other research metrics, such as incorporating community input and membership in planetary health organizations.

### 3.3 Community outreach and advocacy

SCBMCH and SJMC conducted annual cleaning drives and educational programs on plastics, respectively, while KIMS used street plays and flash mobs. FMMC had minimal community outreach [11,13,15,16]. SCBMCH had no community partnerships, and all schools, except SJMC, lacked courses for postgraduate providers or educational materials for patients [11,13,15,16].

### 3.4 Support for student-led planetary health initiatives was varied

SJMC supported initiatives with grants, research opportunities, and events like the 'Tour de Muglur' cycling rally [13]. SCBMCH and KIMS offered limited research opportunities without dedicated funding, while FMMC reported a lack of support for student-led projects [11,15].

### 3.5 Medical school campus sustainability

SCBMCH incorporated a few sustainable practices in new buildings [11]. KIIT University (KIMS) divested from fossil fuels, used renewable energy, and pledged carbon neutrality by 2050 [16]. SJMC sources 90 % of its energy from renewables and promotes eco-friendly practices, and FMMC uses hydroelectric and solar power [13,15]. Despite these efforts, all institutions acknowledged the need for continued progress towards reducing their carbon footprints and achieving sustainability goals.

**Fig 1:**
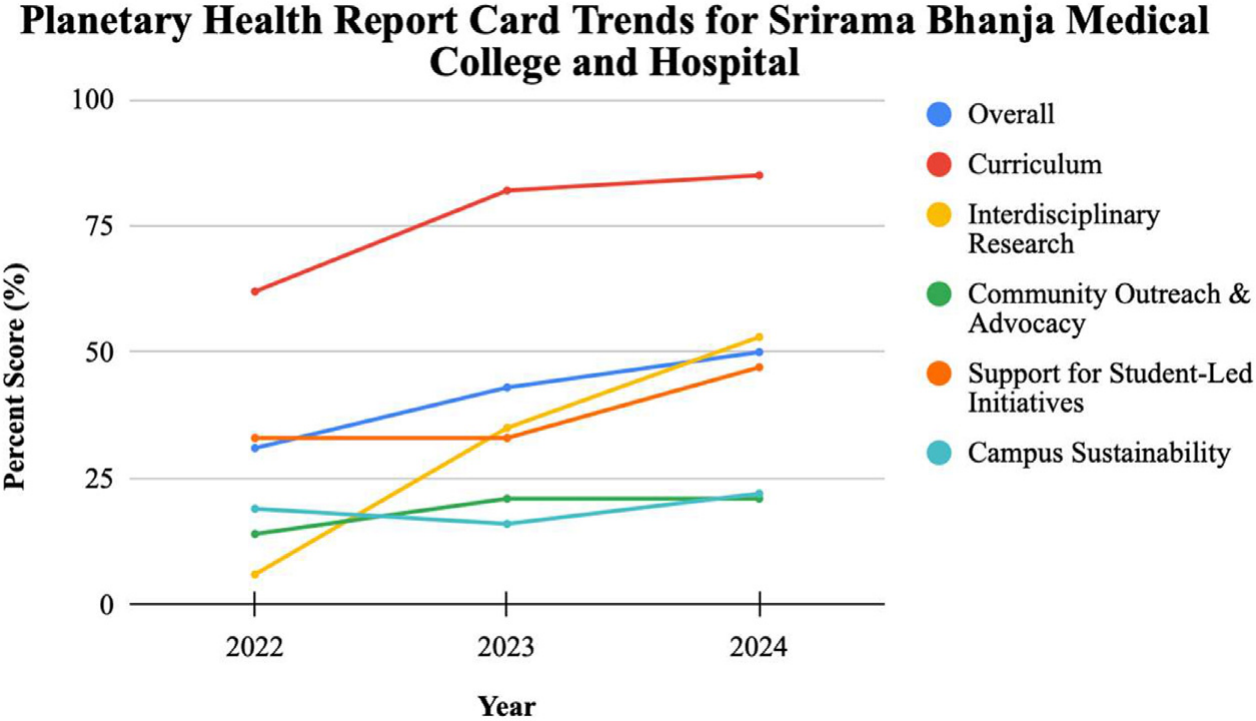
PHRC trends for SCBMCH.

**Fig 2:**
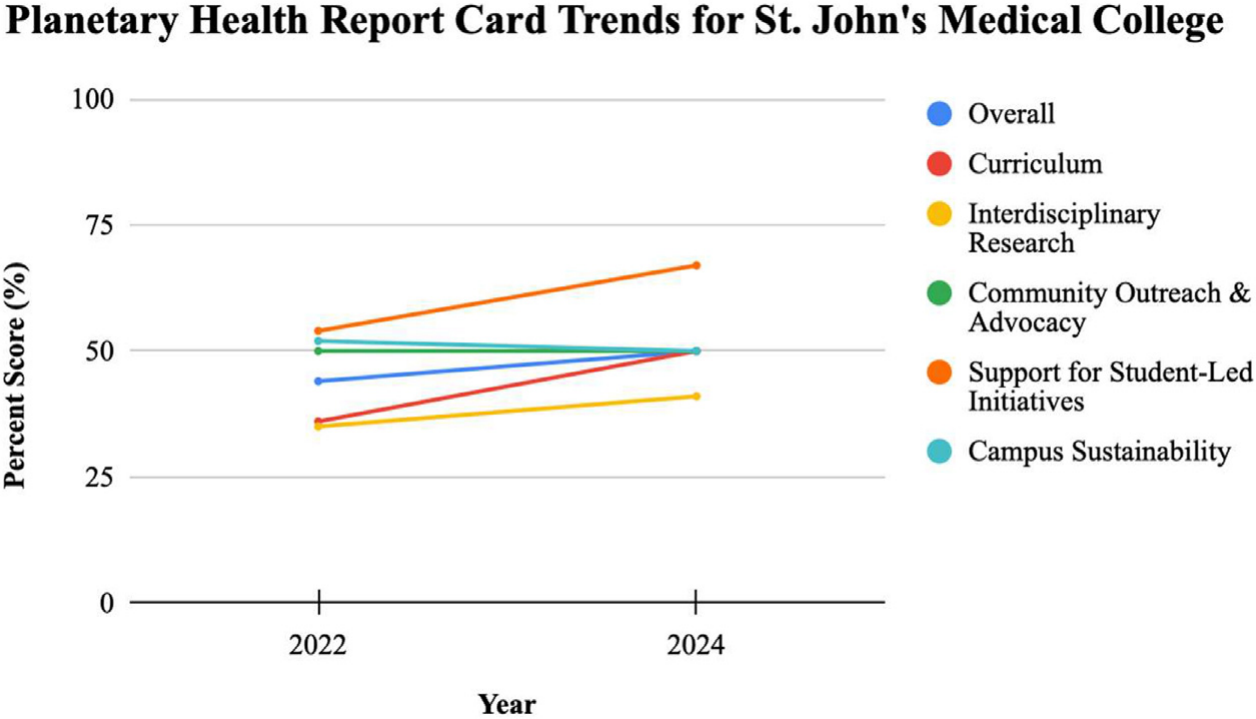
PHRC trends for SJMC.

Fig 1, Fig 2 depict the PHRC trends for SCBMCH and SJMC, respectively. Between 2022 and 2024, overall grades on the PHRC report cards reflected notable progress, with SCBMCH improving from a D to a C, and SJMC rising from a C– to a C. Meanwhile, KIMS received a C+, and FMMC received a D in 2024 [[11], [12], [13], [14], [15], [16]].

## 4 Discussion and recommended actions

The recognition of planetary health within the global academic framework is crucial for adequately preparing healthcare professionals. Despite growing awareness, many healthcare practitioners still have a limited understanding of the health impacts of climate change. In one multinational study, 41 % of physicians and nurses had limited understanding of climate change's health impacts, while 31 % expressed reluctance to educate the public about climate-related matters, and 14 % viewed public education either professionally or personally risky [21]. This underscores the need for a systematic inclusion of planetary health in medical curricula [22].

Effective change, however, requires a systematic approach, including defining the issue, gathering evidence, assessing needs, examining current practices, and implementing and refining interventions [23]. The PHRC initiative exemplifies such an approach, aiming to reform medical education to better equip students for environmentally-related challenges. Since its inception, the PHRC has expanded significantly and encompasses 18 countries and over 150 health professional schools, demonstrating the strong commitment of medical students to drive curriculum improvements worldwide [8,24].

In our cohort, overall grades on the PHRC report cards showed improvement between 2022 and 2024. These metrics provide a framework for medical institutions to assess needs, track progress, and set specific goals to enhance their programs [6].

Future healthcare professionals must be prepared to address the health impacts of environmental change and reduce healthcare’s footprint. This involves promoting planetary health education, supporting research and sustainable practices, and working with vulnerable communities. These efforts are crucial, as climate change disproportionately affects marginalized populations, making this a matter of both health and social justice [25].

To address this mounting concern for human health, the General Medical Council (GMC) in the UK has mandated that graduating physicians should be able to integrate the principles of sustainable healthcare into medical practice [26,27], and the American Medical Association has recommended “incorporating the health implications of climate change into the spectrum of medical education” [28]. To prepare Indian medical students, the NMC, India’s apex regulatory body for medical education and practice, which is responsible for updating curricula to address the nation’s healthcare needs [29] needs to address the impacts of climate change on human and planetary health. Some undergraduate learning outcomes address how environmental factors like air pollution influence disease; however, based on observed gaps in planetary health integration across Indian medical colleges, the following suggestions are proposed for the NMC.

1. **Developing a Planetary Health Coursework or Elective**: Medical schools should introduce dedicated coursework or electives focused on the health impacts of climate change [30].
2. **Establishing Clear Learning Outcomes**: The NMC should establish and surpass graduate outcomes similar to the GMC [26]. It is suggested the NMC also advocate for the integration of "planetary health" into their outcomes in addition to sustainable healthcare because their current competency-based undergraduate curriculum falls short of adequately addressing planetary health [18].
3. **Encouraging Planetary Health-Related Research**: Medical schools should encourage students to explore links between the environment and health through planetary health research, supported by dedicated committees focused on sustainability and planetary health that can also tackle the United Nations Sustainable Development Goals [31].
4. **Centralizing Planetary Health:** Medical schools should develop dedicated, regularly updated websites which can centralize research, events, faculty contacts, and funding opportunities to boost collaboration and visibility across campuses related to climate change and health.
5. **Organizing Outreach Activities**: Medical schools should establish committees to organise outreach activities that raise awareness of climate change’s health impacts, engaging both academic and public audiences to promote understanding.
6. **Making Educational Materials Accessible to Patients:** Medical schools must train students in settings that acknowledge ecological degradation and provide patients with accessible information on environmental health risks [30].
7. **Supporting Student-Led Initiatives**: Medical schools should actively support student-led sustainability initiatives through funding, mentorship, and networking. With limited research groups and supervision at the undergraduate level, it is often students who drive engagement in the emerging field of planetary health. Supporting their efforts is vital to nurturing future healthcare professionals committed to climate and health action.
8. **Improving Campus Sustainability**: Medical schools should lead by adopting sustainable campus practices, such as promoting active travel (e.g. walking and cycling) and using renewable energy. This sets a standard for the healthcare sector and showcases environmental stewardship in action.

## 5 Conclusion

This study reveals disparities in the integration of planetary health among Indian medical schools and underscores the value of tools like the PHRC for assessing, monitoring, and evaluating the needs and progress of planetary health education in medical curricula. To address this, the Ministry of Health and regulatory bodies, such as the NMC, must provide strong and adequate leadership to ensure the vertical integration of planetary health education into medical curricula. This can be achieved through faculty and staff training, the introduction of dedicated courses, the establishment of clear learning outcomes, and support for student-led initiatives. These steps will help to prepare future healthcare professionals with the knowledge and skills needed to address the interconnectedness of human health and planetary well-being, promoting a sustainable healthcare system in India.

## CRediT authorship contribution statement

**Prasoon Pattanaik:** Writing – review & editing, Writing – original draft, Methodology, Formal analysis, Data curation. **Anandita Pattnaik:** Writing – review & editing, Writing – original draft, Validation, Supervision, Project administration, Methodology, Formal analysis, Data curation, Conceptualization.

## Declaration of competing interest

The authors declare that they have no known competing financial interests or personal relationships that could have appeared to influence the work reported in this paper.
